# Study on Differences in 2-AP Synthesis and Metabolism Among Fragrant Rice Varieties

**DOI:** 10.3390/ijms262010102

**Published:** 2025-10-17

**Authors:** Qian Wang, Wuhua Long, Xian Wu, Chaoxin Wu, Yanlong Gong, Zhongni Wang, Susong Zhu

**Affiliations:** 1 Guizhou Rice Research Institute, Guizhou Academy of Agricultural Sciences, Guiyang 550006, China; wqian7821@163.com (Q.W.);; 2 Ministry of Agriculture and Rural Affairs Key Laboratory of Crop Genetic Resources and Germplasm Innovation in Karst Region, Guiyang 550006, China; 3 Institute of Crop Germplasm Resources, Guizhou Academy of Agricultural Sciences, Guiyang 550006, China

**Keywords:** 2-acetyl-1-pyrroline, fragrant rice, synthesis pathways, volatile organic compounds

## Abstract

Fragrant rice is regarded as a premium variety due to its distinctive aroma, delicate texture, and rich nutritional value. This aroma primarily originates from 2-acetyl-1-pyrroline (2-AP), but the metabolic basis of 2-AP remains elusive to this day, and the genetic basis for metabolite accumulation is largely unknown. While several researchers have investigated differences in 2-AP synthesis pathways between fragrant and non-fragrant rice, few studies have examined the 2-AP synthesis pathways in fragrant rice varieties exhibiting 2-AP differences. Therefore, after conducting gene similarity analyses on six fragrant rice varieties, we measured the expression levels of substances and related genes involved in multiple metabolic pathways within the 2-AP synthesis pathway, along with the specific enzyme activities associated with these pathways. Results indicate that XG12 (Guizhou Fragrant Rice Variety) exhibits the highest 2-AP content, yet its efficiency in synthesizing 2-AP is not the highest across any individual metabolic pathway. This finding reveals that among fragrant rice varieties, 2-AP content negatively correlates with *OsBadh2* expression levels and GABA content, while showing no linear correlation with other related substances or metabolic genes. At this point, variations in the major effect gene *OsBadh2* no longer dominate; instead, subtle differences in 2-AP content are jointly determined by numerous minor effect genes and environmental factors. This phenomenon not only resolves apparent contradictions but profoundly illuminates the complex regulatory mechanisms governing 2-AP biosynthesis. 2-AP synthesis represents a dynamic equilibrium process, with different fragrant rice varieties potentially accumulating 2-AP through distinct metabolic pathways. Additionally, this study analyzed the volatile organic compounds (VOCs) of six fragrant rice varieties through metabolomics. Results revealed that DLX, which exhibited the lowest 2-AP content, contained the richest array of aggregated VOCs, indicating no correlation between 2-AP and numerous VOCs. Our findings provide a clear research direction for elucidating the genetic regulatory mechanisms of 2-AP underlying fragrant rice and lay the foundation for technological research aimed at enhancing the aroma of fragrant rice varieties.

## 1. Introduction

The aroma of rice is one of the most important sensory quality characteristics. Fragrant rice, known for its unique aroma, typically commands several times or even ten times the price of ordinary rice. As a result, rice varieties with a rich aroma are highly sought after by consumers in the market [1]. To determine the source of the aroma in fragrant rice, previous studies have investigated the aromatic compounds present in such rice. In 1982, Buttery first identified the aromatic compound 2-AP in fragrant rice [2]. The following year, the team conducted a comparative analysis of 2-AP content in 10 rice varieties, finding that the 2-AP content in 8 fragrant rice varieties was dozens of times higher than that in 2 non-fragrant rice varieties, suggesting that 2-AP is the primary component responsible for the aroma of fragrant rice [3]. Paule and Powers (1989) used GC-MS to detect the content of volatile substances in fragrant rice and non-fragrant rice, while also arranging for two groups of experienced members to conduct sensory evaluations of these volatile substances [4]. The experimental results showed that 2-AP was positively correlated with the fragrance of fragrant rice. Therefore, the view that 2-AP is the main effective component of the fragrance of fragrant rice has gradually gained recognition [5,6].

In rice, the intact BADH2 protein catalyzes the conversion of γ-aminobutyraldehyde (GABald) into γ-aminobutyric acid (GABA). However, in fragrant rice, a mutation in the *OsBadh2* causes loss of protein function, leading to substantial accumulation of its reaction substrate GABald. This substrate then spontaneously cyclizes to form Δ**1**-pyrroline, which serves as the direct precursor for 2-AP synthesis [7,8]. Scholars have confirmed this mechanism, though notably, fully functional BADH2 protein cannot completely inhibit 2-AP production, with trace amounts detectable in some transgenic rice lines overexpressing the gene [9]. It was not until 2024 that research revealed *OsWRKY19* increases 2-AP content by negatively regulating *OsBadh2*, while simultaneously influencing agronomic traits through modulation of yield-related genes [10].

Proline, glutamic acid, and ornithine were first identified as indirect precursor substances in the 2-AP synthesis pathway [11,12]. Schieberle identified two direct synthetic precursors of 2-AP in his studies in 1995 and 1998: methylglyoxal (MG) and Δ**1**-pyrroline. MG acts as an acylating agent in the reaction, and through an acetylation reaction with Δ**1**-pyrroline, 2-AP is formed. Although the direct precursors and indirect precursors for 2-AP synthesis have been identified, the intermediate reactions remain unclear. Huang found a strong positive correlation between MG and 2-AP in *Escherichia coli*. The following year, in their study, they detected the levels of P5C, MG, and Δ**1**-pyrroline-5-carboxylic acid in the callus tissues of three varieties: ‘Tai Nong 71’ (fragrant), ‘Tai Nong 72’ (fragrant), and ‘Tai Nong 67’ (non-fragrant), they measured the content of P5C and MG, the specific enzyme activity of Δ**1**-pyrroline-5-carboxylic acid synthase (P5CS) and ornithine transaminase (OAT), and the expression level of the *OsP5CS* gene in callus tissue. The results showed that the P5C content in ‘Tai Nong 71’ and ‘Tai Nong 72’ was 1.9–2.7 times higher than that in the ‘Tai Nong 67’ variety, while MG was 1.3 times higher. The specific enzyme activities of P5CS and OAT, as well as the expression levels of the *OsP5CS* gene, were also significantly higher than those in ‘Tai Nong 67’ [13]. Therefore, his research indicates that the upregulation of the *OsP5CS* gene contributes to increased P5C content, leading to 2-AP accumulation [14]. Hinge measured the expression levels of genes related to the 2-AP metabolic pathway in the aromatic rice varieties ‘Ambemoar-157’ and ‘Basmati-370’ and the non-aromatic rice variety ‘IR-64’. The results showed that the expression levels of the *OsBadh2* and *Os3-GAPDH* genes were low in aromatic rice, while the transcription levels of the *OsTPI* and *OsP5CS* genes were high. GC-MS analysis also revealed that 2-AP content was higher in aromatic rice than in non-aromatic rice [15]. PCA indicated a strong positive correlation between MG, TPI, and 2-AP.

In the glutamate metabolic pathway, glutamate and P5C can be interconverted via P5CS and P5CDH; proline and P5C can be interconverted via ProDH and P5CR; ornithine can be converted into P5C by OAT, or into putrescine by ODC, which is then catalyzed by DAO to form GABald [16]. Research indicates that simultaneous knockout of both *OsBadh2* and *OsODC* genes significantly increases 2-AP content compared to lines with single *OsBadh2* knockout, without affecting other agronomic traits or rice grain quality [17]. Previous studies have shown that glutamate content increases concurrently with enhanced activity of OAT and ProDH [1]. Although previous studies have shown a positive correlation between P5C levels and 2-AP, the mechanism by which P5C reacts with Δ**1**-pyrroline to form 2-AP remains unclear. In the glycolytic metabolic pathway, G-3-P and DHAP can be interconverted by TPI, with MG as the byproduct. To date, many questions remain unresolved regarding the synthesis pathway of 2-AP, such as the metabolic mechanism by which P5C reacts to produce Δ**1**-pyrroline.

Several scholars have conducted research on the 2-AP synthesis pathway in aromatic rice and non-aromatic rice, but there are few reports on the metabolic differences in 2-AP synthesis between aromatic rice varieties. Therefore, this study mainly focuses on whether the differences in 2-AP production between aromatic rice varieties with the same *OsBadh2* allele mutation grown in the same environment are influenced by differences in metabolic levels in the metabolic pathway, which affect its synthesis efficiency.

## 2. Results and Analysis

### 2.1. Analysis of the Genetic Background of Rice

**Figure 1:**
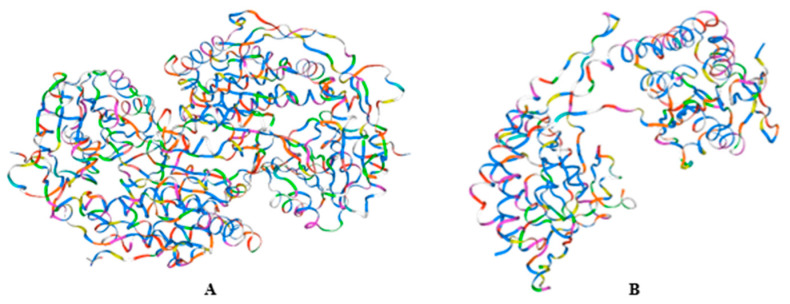
Protein structure of wild-type *Badh2* (**A**) and *badh2-E7* mutants (**B**) protein structure.

We selected six fragrant rice varieties to elucidate the reasons for the differences in aroma between them. These selected varieties primarily share the same allele type characterized by an 8 bp deletion and a 3 bp mutation in the 7th exon of the *Osbadh2* gene (Table 1). This allele type causes premature translation termination, resulting in a nonfunctional truncated protein (Figure 1). The loss of this functional protein leads to the accumulation of 2-AP, thereby producing the aroma [7,17,18].

**Figure 2:**
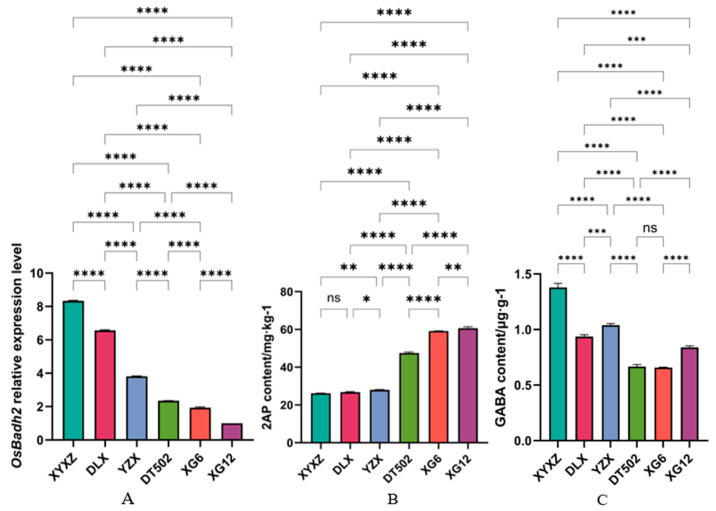
*OsBadh2* expression levels (**A**) and 2-AP (**B**) and GABA (**C**) content. **Note**: ‘ns’ no significant difference, ‘*’ *p* < 0.05, ‘**’ *p* < 0.01, ‘***’ *p* < 0.001, ‘****’ *p* < 0.0001.

Fluorescent qPCR was used to detect changes in the mRNA levels of *OsBadh2*. The results showed that there was no significant difference between DT502 and XG6, while significant differences were observed among the remaining varieties. The highest expression level was observed in XYXZ, while the lowest was in XG12 (Figure 2A). GC-MS analysis of 2-AP content showed that XG12 had the highest 2-AP content, while XYXZ had the lowest (Figure 2B). Previous studies have demonstrated that *OsBadh2* expression levels negatively correlate with 2-AP content, and 2-AP content negatively correlates with GABA content (Figure 2C) [19]. The results of this study are consistent with these findings.

**Figure 3:**
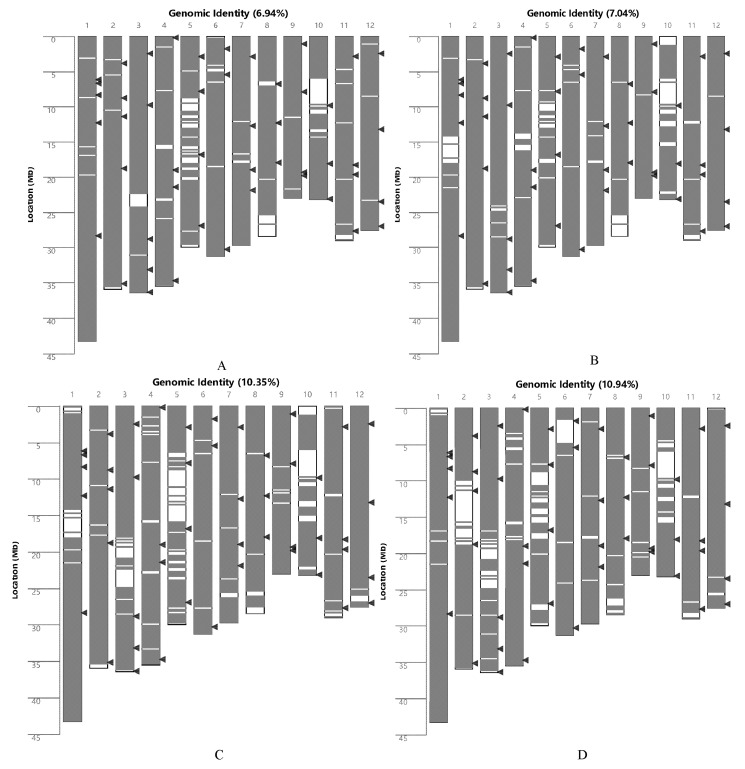

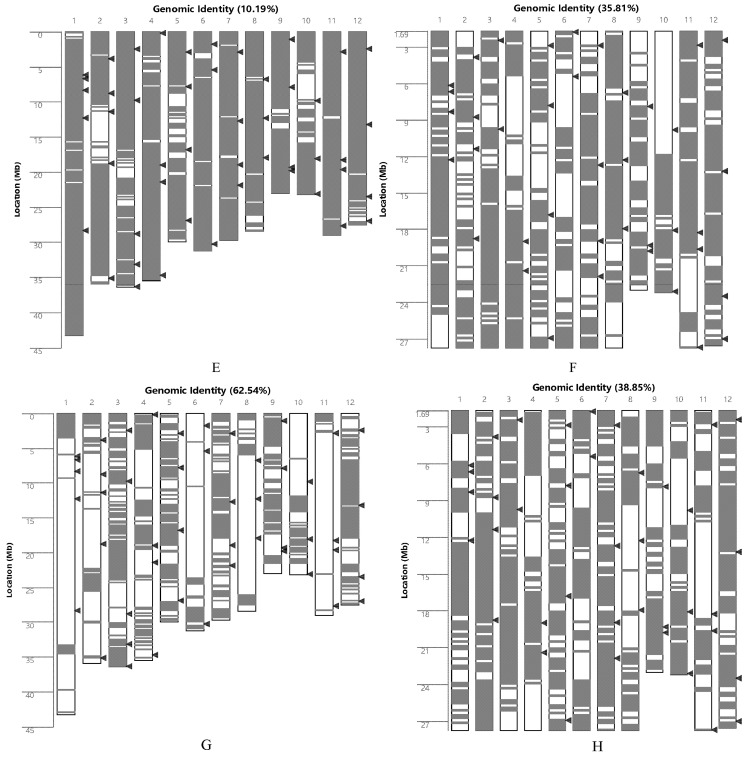

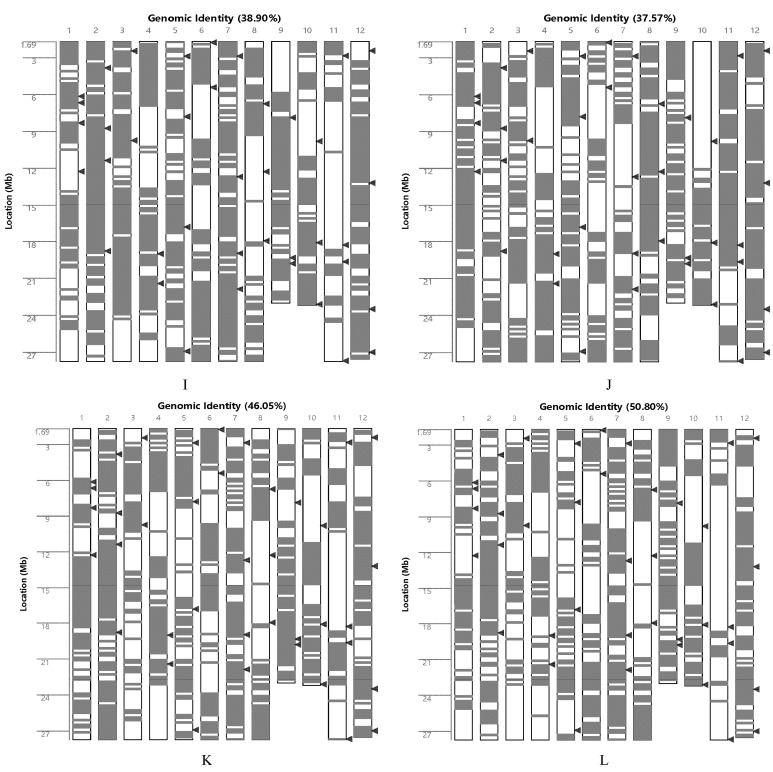

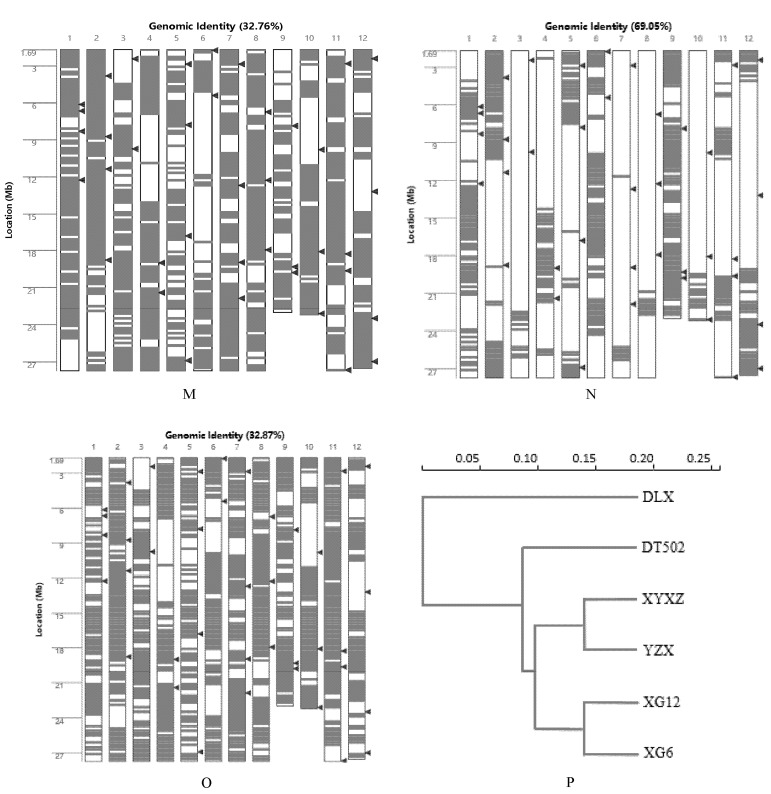
Genomic similarity analysis. (**A**–**E**) is the genomic similarity between DLX and DT502, XG6, XG12, XYXZ, and YZX, respectively; (**F**–**I**) is the genomic similarity between XG6, DT502, XG12, XYXZ, and YZX, respectively; (**J**–**L**) is the genomic similarity between XG12, DT502, XYXZ, and YZX, respectively (**J**–**L**) is the genomic similarity between XG12 and DT502, XYXZ, and YZX, respectively; (**M**,**N**) is the genomic similarity between YZX and DT502 and XYXZ, respectively; (**O**) is the genomic similarity between XYXZ and DT502, respectively. (**P**) Clustering of genetic background relationships among six varieties. **Note:** The triangles in the figure indicate the locations of the 48 national standard SSR markers in rice, and the red color indicates that the region is not similar.

We analyzed the test materials using a 40 K rice gene chip. The results showed that the genomic similarity between DLX and DT502, XG6, XG12, XYXZ, and YZX was 6.94%, 7.04%, 10.35%, 10.94%, and 10.19%, respectively (Figure 3A–E). The genomic similarities between XG6 and DT502, XG12, XYXZ, and YZX were 35.81%, 62.54%, 38.85%, and 38.90%, respectively (Figure 3F–I); the genomic similarities between XG12 and DT502, XYXZ, and YZX were 37.57%, 46.05%, and 50.80% (Figure 3J–L); The genomic similarity between YZX and DT502 and XYXZ was 32.76% and 69.05% (Figure 3M,N); the genomic similarity between XYXZ and DT502 was 32.87% (Figure 3O). Cluster analysis grouped XG6 and XG12 into one category. These two varieties were bred from conventional rice Xi Li Gong Mi mutant strains from the Rongjiang area of Qiandongnan Prefecture, Guizhou Province. YZX and XYXZ were clustered together because XYXZ’s maternal parent was YZX. DT502 and DLX were each grouped into separate categories (Figure 3P).

Based on the above analysis results, the intensity of the fragrance of fragrant rice is not only influenced by different allelic mutations of the *OsBadh2* gene but also varies to different degrees among 2-APs of the same *OsBadh2* allele type grown under the same conditions. This may be due to the influence of the genetic background of the variety itself.

### 2.2. Analysis of the Metabolic Pathway for the Production of 2-AP from Putrescine and Spermine

Due to the highly complex metabolic pathways of 2-AP, researchers have focused more on investigating its synthesis pathways, which are currently categorized into two potential routes. First, putrescine and spermine are catalyzed by DAO and PAO, respectively, to form GABald. GABald then forms Δ^1^-pyrroline without betaine aldehyde dehydrogenase involvement. Δ^1^-pyrroline reacts with MG via enzymatic or non-enzymatic pathways to produce 2-AP [20]. In fragrant rice, 2-AP content negatively correlates with GABA levels, while wild-type plants exhibit higher GABA content [15,21].

**Figure 4:**
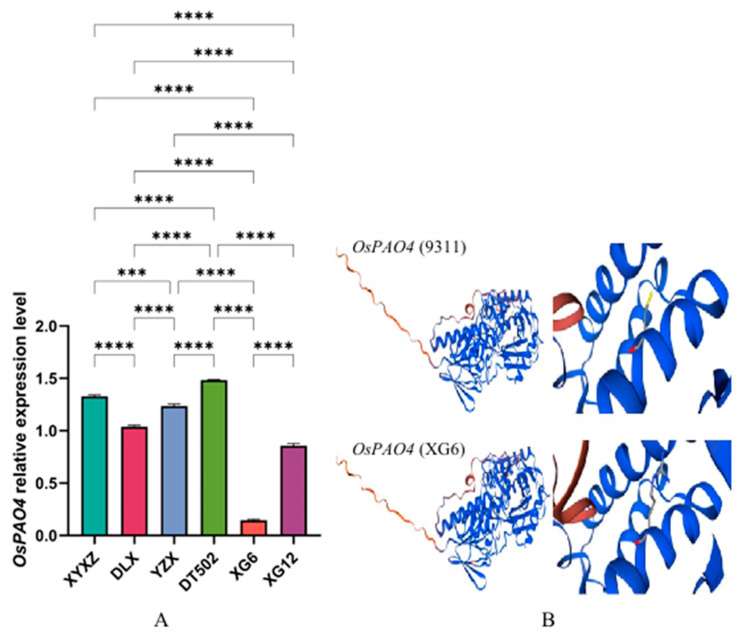
*OsPAO4* expression levels in rice (**A**); Comparison of 9311 and XG6 *OsPAO4* proteins (**B**). **Note:** ‘***’ *p* < 0.001, ‘****’ *p* < 0.0001.

We detected GABA levels in six varieties via GC-MS, finding results positively correlated with *OsBadh2* expression levels and negatively correlated with 2-AP content (Figure 2C). In the first potential pathway for 2-AP synthesis, we learned that polyamines (PAs) include putrescine (Put), spermine (Spm), and spermidine (Spd), with putrescine serving as the central metabolite in polyamine biometabolism [22]. The polyamine degradation pathway involves the catalyzed conversion of diamines or polyamines (PAs) into GABA by diamine oxidase (DAO4) and polyamine oxidase (PAO4), respectively. Through CDS sequencing, we found that the *OsDAO4* CDS sequence in all six varieties was identical to 9311. In XG6, the *OsPAO4* gene CDS sequence exhibited a T-to-C mutation at the 556th base (Table 2), resulting in a codon change from TGT (cysteine) to CGT (arginine). *OsPAO4* expression levels in XG6 were significantly lower than in the other five varieties (Figure 4A), and the protein structure also changed (Figure 4B). Therefore, in the pathway converting spermine (spermin) to GABald, XG6 exhibits lower metabolic efficiency than the other five varieties.

### 2.3. Analysis of the 2-AP Metabolic Pathway for Proline, Glutamic Acid, and Ornithine

**Figure 5:**
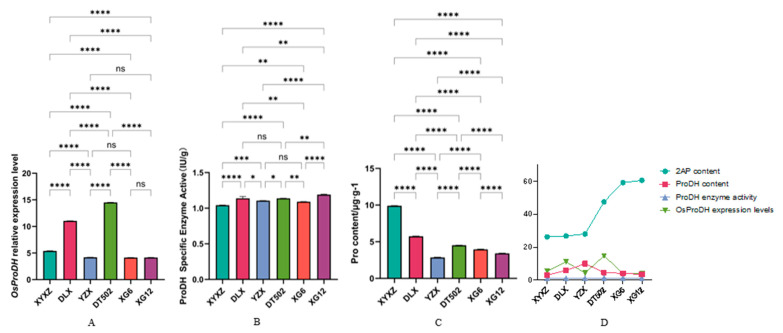
Expression levels of *OsProDH* (**A**); Enzyme activity of ProDH (**B**); Content of Proline (**C**); ProDH content, expression levels, and enzymological activity exhibit a linear relationship with 2-AP (**D**). **Note**: ‘ns’ no significant difference, ‘*’ *p* < 0.05, ‘**’ *p* < 0.01, ‘***’ *p* < 0.001, ‘****’ *p* < 0.0001.

Another potential pathway for 2-AP synthesis involves proline catalyzed by proline dehydrogenase (ProDH), ornithine catalyzed by ornithine transcarbamoylase (OAT), and glutamate catalyzed by pyrroline-5-carboxylic acid synthase (P5CS), all yielding P5C. P5C is then regenerated into Δ^1^-pyrroline, which undergoes acylation with MG to ultimately yield 2-AP [13,23,24]. First, in the pathway catalyzed by ProDH for proline oxidation to P5C, we examined the coding sequences of *OsProDH* from six varieties, with results consistent with the wild type. Analysis of *OsProDH* expression levels revealed that DT502 exhibited significantly higher expression than other varieties, DLX showed significantly lower expression than DT502 but significantly higher than the remaining varieties, XYXZ displayed significantly lower expression than DT502 and DLX but significantly higher than the other varieties, while YZX, XG6, and XG12 all showed significantly lower expression than DT502, DLX, and XYXZ. No significant differences were observed among these three varieties (Figure 5A). Furthermore, we measured the specific activity of the ProDH enzyme. Results showed that XG12 was significantly higher than other varieties, XYXZ was significantly lower than the remaining varieties, DLX and DT502, as well as YZX and XG6, showed no significant differences, while significant differences existed among the remaining varieties (Figure 5B). We simultaneously measured proline content in the six varieties (Figure 5C). XYXZ exhibited the highest proline content (9.9 µg/g), significantly higher than the other varieties. DLX’s proline content (5.74 µg/g) was significantly higher than the remaining four varieties except XYXZ. YZX exhibited the lowest proline content (2.87 µg/g), significantly lower than all other varieties. DT502’s proline content (4.51 µg/g) was significantly lower than both XYXZ and DLX. XG6 and XG12 contained 3.97 µg/g and 3.42 µg/g proline, respectively. Based on these results, we observed no proportional increase in proline content, enzyme activity, or gene expression levels among the six aromatic rice varieties in relation to 2-AP content (Figure 5D). This inconsistency indicates that 2-AP accumulation likely undergoes complex regulation by multiple factors (proline content, enzyme activity, gene expression) rather than simple linear dependence.

**Figure 6:**
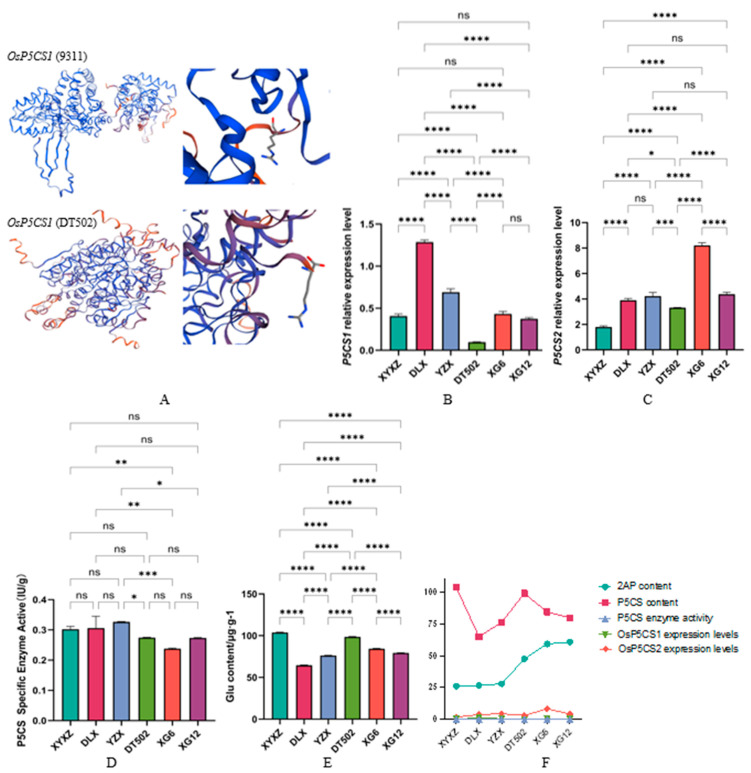
Protein Structure of *OsP5CS1* in 9311 and DT502 (**A**); Expression levels of *OsP5CS1* (**B**); Expression levels of *OsP5CS2* (**C**); Enzyme activity of P5CS (**D**); Content of glutamic acid (**E**); P5CS content, expression levels, and enzymological activity exhibit a linear relationship with 2-AP (**F**). **Note:** ‘ns’ no significant difference, ‘*’ *p* < 0.05, ‘**’ *p* < 0.01, ‘***’ *p* < 0.001, ‘****’ *p* < 0.0001.

Second, glutamate is catalyzed by P5CS to form P5C. *OsP5CS1* is responsible for basal glutamate synthesis, while *OsP5CS2* is primarily induced under stress conditions (drought, salt) [25]. In this pathway, sequencing of the *OsP5CS1* and *OsP5CS2* coding sequences revealed distinct nucleotide variations in *OsP5CS1* across five materials except DLX. Notably, DT502 exhibits a 1 bp insertion (T) at position 192 (Table 3), causing premature translation termination and altering protein structure (Figure 6A). We also measured the expression levels of *OsP5CS1* and *OsP5CS2* across six varieties. Due to premature termination in DT502, its *OsP5CS1* expression was the lowest (Figure 6B), significantly lower than all other varieties. DLX exhibited significantly higher expressions than all other varieties. YZX showed significantly lower expression than DLX but significantly higher than the remaining varieties. No significant differences were observed among XYXZ, XG6, and XG12. *OsP5CS2* expression levels (Figure 6C) showed XG6 was significantly higher than other varieties. XYXZ was significantly lower than the other varieties. No significant differences were observed among DLX, YZX, and XG12, all of which were significantly higher than XYXZ and DT502. Additionally, we measured the specific activity of the P5CS enzyme (Figure 6D). Results showed that XYXZ and DLX were significantly higher than XG6, while YZX was significantly higher than DT502, XG6, and XG12. No significant differences were found among the remaining varieties. We simultaneously measured glutamic acid content in the six varieties (Figure 6E). The highest content was observed in XYXZ (103.52 µg/g), followed by DT502 (98.69 µg/g), XG6 (84.24 µg/g), XG12 (79.59 µg/g), and YZX (76.18 µg/g), with the lowest being DLX (64.74 µg/g). Significant differences in glutamic acid content were observed among all varieties. Based on these results, we found no proportional increase in glutamic acid content, enzyme activity, or gene expression levels among the six fragrant rice varieties in relation to 2-AP content (Figure 6F). This suggests that glutamic acid may be diverted to alternative nitrogen metabolism pathways (e.g., GABA or TCA cycle) rather than entering the aroma metabolic pathway, potentially because it cannot be efficiently converted to P5C and is instead stored as an osmotic regulator.

**Figure 7:**
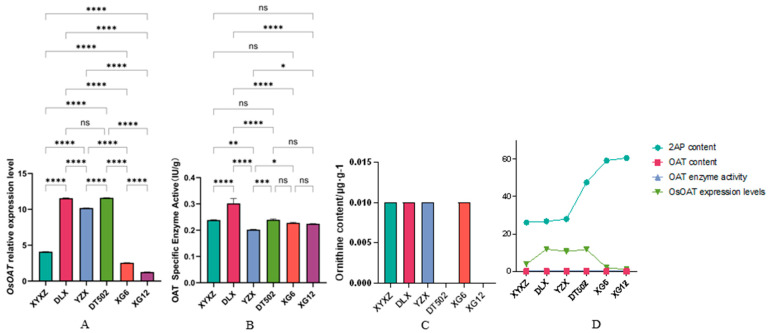
Expression levels of *OsOAT* (**A**); Enzyme activity of OAT (**B**); Content of ornithine (**C**); OAT content, expression levels, and enzymological activity exhibit a linear relationship with 2-AP (**D**). **Note:** ‘ns’ no significant difference, ‘*’ *p* < 0.05, ‘**’ *p* < 0.01, ‘***’ *p* < 0.001, ‘****’ *p* < 0.0001.

Finally, ornithine is catalyzed by OAT to produce P5C; no CDS variants were identified in this pathway. Analysis of *OsOAT* expression levels revealed no significant difference between DLX and DT502, though both were significantly higher than other varieties. YZX was significantly higher than XYXZ, XG6, and XG12. XYXZ was significantly higher than XG6 and XG12. XG12 was significantly lower than the other varieties (Figure 7A). Additionally, we measured the specific activity of the OAT enzyme, which showed that DLX had significantly higher enzyme activity than the other varieties. No significant differences were observed among XYXZ, DT502, XG6, and XG12, but all were significantly higher than YZX (Figure 7B). We simultaneously measured ornithine content in the six varieties. Results showed undetectable levels in DT502 and XG12, while the remaining four varieties contained 0.01 µg/g (Figure 7C). Notably, DT502, which showed undetectable ornithine levels, exhibited the highest *OsOAT* expression (Figure 7D). This non-linear relationship may result from competitive pathway effects, where ornithine is converted to putrescine by ODC, entering the putrescine metabolic pathway and thereby offsetting OAT consumption.

### 2.4. Analysis of Volatile Metabolites in Rice Aroma

**Figure 8:**
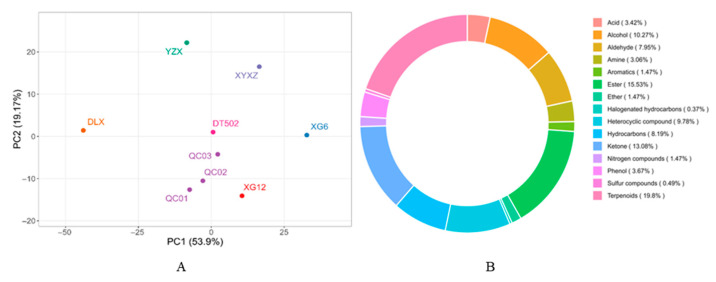
Principal component analysis of grain metabolites. Each variety is marked with a small dot of a different color (**A**); Distribution of volatile metabolite subclasses. The proportions are shown for 15 subclasses (**B**).

**Figure 9:**
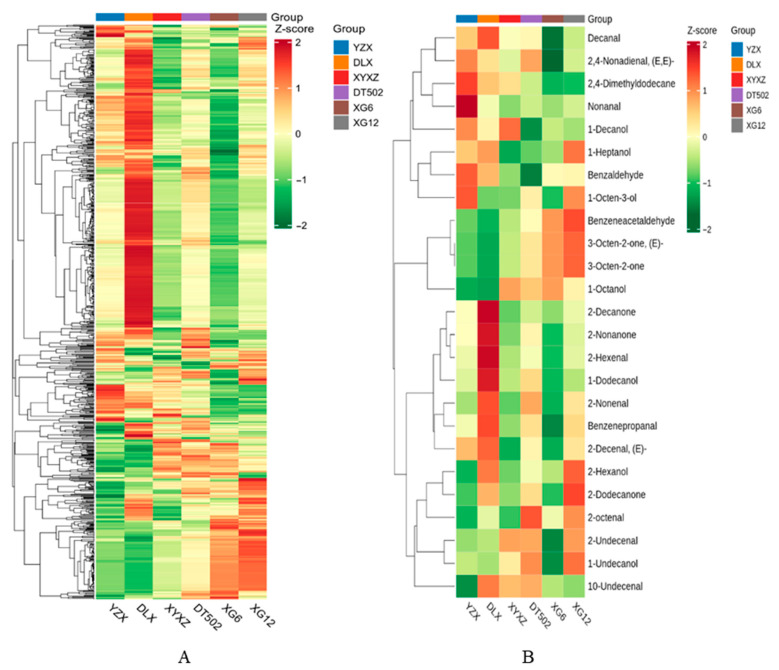
All VOCs clustering heatmap (**A**); Heatmap of key VOCs in rice (**B**).

In addition to 2-AP, we detected all volatile compounds in rice grains and performed principal component analysis (PCA) on the metabolome data (Figure 8A). Principal Component 1 (PC1) and Principal Component 2 (PC2) explained 53.9% and 19.17% of the variance, respectively. The results showed that each variety was clearly separated along PC1, indicating significant differences in volatile metabolite composition among the six varieties. Over 300 rice-related volatiles have been reported, predominantly categorized as hydrocarbons, aldehydes, ketones, esters, acids, alcohols, and heterocyclic compounds. Among these, aldehydes, ketones, and alcohols are the primary factors influencing rice grain volatiles [26]. We grouped the detected volatiles into 15 categories (Figure 8B). Previous studies indicate that nonanal, decanal, 1-octanol, 1-heptanol, 1-octen-3-ol, and 1-dodecanol are key aromatic VOCs in rice [15]. This study further elucidated potential correlations among volatile aroma compounds in the six varieties by constructing a correlation heatmap (Figure 9A). We further screened other synergistic aroma compounds in rice, excluding 2-AP, from all volatile substances. These included numerous aldehydes, along with some alcohols and ketones (Figure 9B). Aldehydes primarily contribute to the green aroma background. Ketones synergize with aldehydes in green aromas, preventing aldehyde aromas from sounding monotonous. Ketones mainly provide milky and fruity aroma backgrounds, playing a key role in enhancing aroma quality. When coexisting, they undergo complex interactions that layer different aromas, releasing appetizing fragrances.

Six varieties were cultivated under identical conditions, yet significant variations in VOCs richness correlations were observed among them, indicating that genetic factors play a dominant role. Results indicate that DLX exhibits strong correlations with these volatile compounds and displays relatively abundant VOCs. This suggests DLX possesses unique metabolic pathways in the accumulation, synthesis pathways, or regulatory mechanisms of aromatic metabolites. Enhanced activity in the lipoxygenase (LOX) pathway, shikimic acid pathway, or amino acid degradation pathway may contribute to the synergistic accumulation of more aromatic compounds. Furthermore, the intensity of fragrant rice aroma is influenced not only by different mutation types of the *OsBadh2* but also by the genetic background of the fragrant rice variety itself [27]. This is precisely why DLX was classified into a separate cluster in the analysis. This variety has become one of Guizhou Province’s uniquely representative fragrant rice cultivars. Despite its relatively low 2-AP content, it synthesizes a diverse array of volatile organic compounds (VOCs), resulting in a rich aroma and flavor profile.

## 3. Discussion

Aroma is one of the most important edible quality traits of rice, making aromatic rice varieties highly favored by consumers in the market [28]. Some scholars have proposed that the intensity of aroma in fragrant rice is influenced not only by different mutation types of the *OsBadh2* gene but also by the genetic background of the fragrant rice varieties themselves [27]. In this study, two groups of varieties showed greater than 60% gene similarity: XYXZ and YZX, as well as XG6 and XG12. The 2-AP between these pairs exhibited differences, though not extremely significant, indicating that genetic background does indeed exert a certain degree of influence on 2-AP.

Previous studies have also shown that in aromatic rice, when 2-AP accumulates in large quantities, the levels of proline, glutamic acid, and ornithine, along with their corresponding gene expression levels and enzyme activities, are higher than in non-aromatic rice [9,29]. According to our findings, among the six fragrant rice varieties, XYXZ exhibited the lowest 2-AP content but the highest levels of proline, glutamic acid, and ornithine. Only the expression level of *OsP5CS2* was the lowest in this variety, while the remaining indicators were at intermediate levels. Previous studies have attempted to increase 2-AP content in aromatic rice through exogenous application of proline, Mn, and GABA [30,31,32]. Despite having the highest proline content, XYXZ exhibited the lowest 2-AP content. This discrepancy likely stems from these amino acids serving as multifunctional molecules within plants. Beyond acting as precursors for 2-AP, they participate in stress resistance, drought and salt tolerance, osmotic regulation, and nitrogen storage. Under stress conditions, plants may prioritize synthesizing these amino acids for defense mechanisms, allocating only a portion to the 2-AP synthesis pathway.

Second, among varieties with 2-AP content slightly higher than XYXZ, DLX exhibited the highest expression levels of *OsP5CS1* and *OsOAT* genes, as well as the highest specific activities of ProDH, P5CS, and OAT enzymes, while displaying the lowest glutamic acid content. Similarly, YZX, which also had intermediate 2-AP content, exhibited the highest glutamic acid reductase activity and the lowest ornithine transcarbamylase activity and proline content, with all other indicators falling within the intermediate range. Research indicates that alternating wet and dry treatments during the heading stage of fragrant rice can enhance the enzymatic activities of ProDH, P5CS2, and DAO, thereby increasing 2-AP content [33]. Based on enzyme activity variations, 2-AP synthesis involves multiple enzymes. The natural activity of these enzymes may differ among fragrant rice varieties. However, the variety with the highest enzyme activity did not exhibit the highest 2-AP content, suggesting that DLX enzymes may not function efficiently or that other rate-limiting steps exist.

Another variety, DT502, exhibited intermediate 2-AP content with the highest expression levels of both *OsProDH* and *OsOAT*. However, due to a 1 bp base insertion in the coding region of *OsP5CS1* causing premature translation termination, its expression level was the lowest among the six varieties, while all other indicators remained at intermediate levels. Previous studies employing gene editing techniques to overexpress the *OsP5CS* gene in fragrant rice resulted in substantial 2-AP accumulation [14,34]. However, DT502 exhibits lower *OsP5CS1* expression yet higher 2-AP content than XYXZ, which has the highest *OsP5CS1* expression. From the perspective of gene expression levels, the relative expression of genes involved in the 2-AP synthesis pathway is not the sole determinant of 2-AP content when all plants carry the same loss-of-function mutation (*OsBadh2-E7*). A gene exhibiting high expression levels but producing only moderate 2-AP yields may indicate that its mutant protein retains residual activity or that precursor supply is insufficient.

Variety XG6, which has the second-highest 2-AP content after XG12, exhibits the lowest specific enzyme activity for glutamic acid and the highest expression level of *OsP5CS2*. Its *OsOAT* expression level is significantly higher than that of the lowest-expressing variety, XG12. Concurrently, despite exhibiting the lowest expression levels among the six varieties due to a 1 bp mutation in the coding region of *OsPAO4*, its 2-AP content remained substantial. This suggests that another pathway may serve as the primary mechanism for efficient 2-AP accumulation in this variety.

Most notably, the variety XG12 with the highest 2-AP content did not exhibit the highest levels of proline, glutamic acid, or ornithine, nor did it show the highest gene expression levels. While it possessed the highest proline P5CS enzyme activity, its P5CS and OAT enzyme activities were not the highest. Therefore, we infer that if P5C in XG12 primarily originates from any single highly efficient synthesis pathway, the levels of upstream compounds in other pathways may be decoupled from 2-AP. Additionally, P5C metabolism exhibits dynamic equilibrium, suggesting that P5C in XG12 may undergo further metabolism via P5CR or P5CDH. Even P5C itself may independently enhance 2-AP synthesis through one pathway, such as DAO or γ-aminobutyraldehyde dehydrogenase (ABADH) potentially contributing to 2-AP production. Variations in their activities could disrupt the correlation between proline/glutamic acid/ornithine and 2-AP formation, thereby reducing dependence on these precursors. Different aromatic rice varieties may accumulate 2-AP through distinct metabolic pathways. This flexibility obscures the correlation between single gene expression or enzyme activity and 2-AP content.

Previous studies have primarily focused on macro-level comparisons between aromatic and non-aromatic rice varieties. Consistent findings indicate that aromatic rice generally exhibits higher levels of 2-AP content, precursor substance content, enzyme activity, and gene expression compared to non-aromatic rice [35]. This consistency primarily stems from the presence or absence of 2-AP. When comparing these two distinct groups—fragrant and non-fragrant rice—the decisive factor is the functional loss of the major effect gene (*OsBadh2*). At this level, all indicators consistently show “fragrant rice > non-fragrant rice,” a pattern determined by the switch effect of the major effect gene. As long as this switch is “on” (Badh2 inactivated), the system possesses the fundamental conditions for 2-AP production. However, when comparing 2-AP differences among fragrant rice varieties in this study, the trends of these indicators diverged. Since the highest/lowest values for each indicator appeared across six distinct varieties, it can be concluded that no linear growth relationship exists among these indicators within fragrant rice. At this point, the primary distinction lies in the relative abundance of 2-AP. When all compared varieties are fragrant rice with the “switch” already activated (*OsBadh2-E7*), differences in major genes cease to be the primary factor. At this stage, subtle variations in 2-AP content are determined by numerous minor-effect genes, leading to the non-linear changes observed among the indicators. This phenomenon not only resolves apparent contradictions but profoundly reveals the complex regulatory mechanisms governing 2-AP biosynthesis.

## 4. Materials and Methods

### 4.1. Material Reagents

The experimental materials included the Hunan-bred varieties YZX and XYXZ, whose rice possesses excellent quality meeting the national first-class premium rice variety standards; the variety DT502, which has won gold awards for premium rice at the World Expo, from the Ministry of Agriculture, and from the Yunnan Provincial Government; DLX, a Guizhou-bred variety, has been honored with China’s Top Ten Rice Gold Awards; XG6 and XG12, also bred in Guizhou, were recognized as premium products at the 2005 National High-Quality Rice Expo. To eliminate the influence of various environmental factors, in 2024, these varieties were cultivated under uniform conditions in paddy fields at the Guizhou Rice Research Institute (106.660° E, 26.499° N). Upon maturity, six plants per variety were harvested for subsequent experiments (Table 4).

### 4.2. Chip Detection

**Figure 10:**
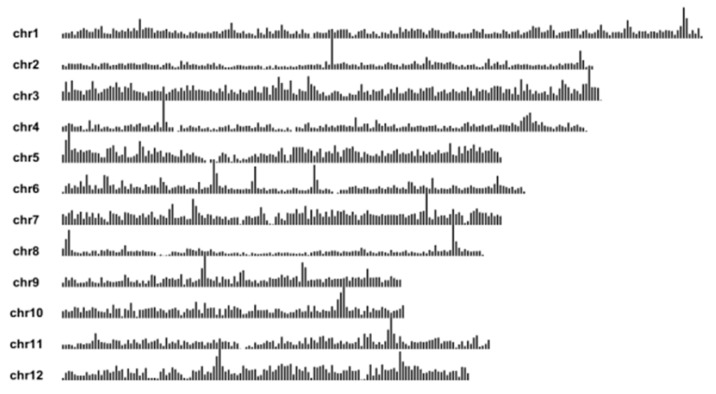
Schematic distribution of all SLYm1R loci across the genome.

The SLYm1R high-density rice genome-wide SNP microarray was used, designed based on Illumina’s chip fabrication technology, and contained 31,753 high-quality loci, of which 31,704 had coordinate position information and the remaining 49 were other probes (Figure 10). The loci screening criteria were:

1. GenTrain Score (The SNP cluster quality) > 0.6;
2. Parental genotypes are pure (markers in which the majority of parental samples are heterozygous will be judged to be of poor quality, usually less than 5% heterozygosity is allowed);
3. The number of genotypic deletions is less than 20% (except for Indel markers);
4. High rate of correct typing.

DNA extraction, whole genome amplification, DNA purification, DNA resuspension, DNA denaturation, DNA hybridization to the microarray, single base extension and staining, microarray scanning, and data typing in specific assays were carried out with reference to the method of [36].

### 4.3. Genomic Similarity

The rice genome was divided into 1874 bins according to 200 KB as a block, and if there was one SNP difference in this bin, the two samples were considered to be different in this block, which avoids the error brought by the high genetic similarity of the two samples due to the existence of discrete differences in the whole genome range, and the genome similarity is more reflective of the real genetic similarity of the two samples. Genomic Identity = (Total number of bins − Differential number of bins)/Total number of bins × 100%.

### 4.4. Gene Expression Level Detection

RNA was extracted from leaf tissue using the RNApure Plant Kit (DNase I) from CWBIO Company. The extracted RNA was reverse transcribed into cDNA using the reverse transcription kit from CWBIO Company. Gene expression analysis was performed using CFX96 Bio-Rad RT−qPCR. The methods for cDNA synthesis and RT−qPCR were the same as those described by Shi [37]. The specific genetic information is presented in Table 5.

### 4.5. Sample Preparation and Content Detection of 2-AP, GABA, Proline, Glutamic Acid, and Ornithine

Standards: 2-Acetyl-1-pyrroline (Weiye Metrology, CAS: 85213-22-5, concentration of 1000 µg/mL), amino acid mixed standard: (glutamic acid, proline), ornithine hydrochloride (Shanghai Amperex CAS: 3184-13-2 purity: 99.9%), γ-amino butyric acid (Shanghai Yuanye CAS: 56-12-2 purity: 99.2%). Reagents: ethanol (CNW, chromatographic purity), formic acid (chromatographic purity), acetonitrile (HPLC), experimental water.

Plotting standard curve: The 2-AP standard was diluted with ethanol to the standard series concentrations of 10, 20, 50, 100, 200, 500, 1000, 2000, 5000, 10,000, 20,000 ng/mL, respectively, and performed using a gas chromatography-tandem mass spectrometry system (Agilent 8890 + 7010 B).

Sample extraction: Weigh about 1.0 g of the sample in a 10 mL centrifuge tube, weigh it precisely, and place the sample on dry ice during the weighing process to avoid volatilization of the aroma. Add 3 mL of anhydrous ethanol to the sample, tighten the screw cap of the centrifuge tube immediately and seal it with a sealing film, vortex for 30 S, ultrasonic at 68 °C for 2 h, centrifuge at 10,000 r/min and 4 °C for 10 min, take the supernatant and pass it through a 0.22 µm filter membrane, performed using a gas chromatography-tandem mass spectrometry system (Agilent 8890 + 7010 B).

Chromatographic conditions: Column: HP-5MS (30 m × 0.25 mm × 0.25 µm); Inlet temperature: 250 °C Flow rate: 1.mL/min; Injection volume: 1 µL; Temperature increase program: Maintain at 45 °C for 1 min, then increase to 100 °C at a rate of 8 °C/min, and then increase to 250 °C at a rate of 50 °C/min, maintaining for 1 min.

Electron bombardment source: 70 eV; Ionization mode is electron bombardment ion source: EI source; Interface temperature 250 °C; Ion source temperature 250 °C;

Plotting standard curve: The 4 standards were prepared into 1000 nmol/mL standard reserve solution with water, and stored temporarily in the refrigerator at 4 °C. Then dilute into a series of standard solutions of 0.01, 0.05, 0.1, 1, 5, 10, 20, 50 nmol/mL with 0.1% formic acid water, and use it now.

Sample extraction: weigh about 1.0 g of sample, add 0.1% formic acid-water solution 5 mL, vortex mixing and ultrasonic extraction for 30 min, centrifugation at 5000 RPM for 5 min, take the supernatant through the membrane, dilution with a 0.1% formic acid aqueous solution, it was placed into the liquid chromatography-mass spectrometry system (QTRAP 5500, AB Sciex, Concord, ON, Canada).

Chromatographic conditions: Column: AgilentPoroshell120HILIC-Z (2.7 µm 3.0 × 150 mm).

Column temperature: 30 °C; Flow rate: 0.5 mL/min; Injection volume: 5 µL; Phase A: 0.1% formic acid-water solution, Phase B: acetonitrile; Gradient elution.

Mass spectrometry parameters: Ionization mode: ESI positive ion mode.

Scan type: MRM.

### 4.6. Enzyme Assays for PRODH, P5CS, and OAT

a. Sample Extraction: Tissue homogenization is performed at a 10% ratio, equivalent to 1 g of tissue plus 9 mL of homogenization buffer. The tissue mass (g) to extraction buffer volume (mL) ratio is 1:9. The homogenization buffer must be PBS with a concentration of 0.01 M and a pH controlled between 7.2 and 7.4.

b. Standard Dilution: Dilute the undiluted standards provided in the kit as follows: ProDH assay: 10 IU/L, 20 IU/L, 40 IU/L, 80 IU/L, 160 IU/L in small tubes. P5CS assay: Dilute in small tubes at concentrations of 1.5 IU/L, 3 IU/L, 6 IU/L, 12 IU/L, and 24 IU/L. OAT assay: Dilute in small tubes at concentrations of 1 IU/L, 2 IU/L, 4 IU/L, 8 IU/L, and 16 IU/L.

c. Sample Addition: Prepare blank wells (blank control wells without sample or enzyme-labeled reagent, but follow all other steps identically), standard wells, and sample wells. Accurately dispense 50 µL of standard solution into the standard wells on the coated microplate. For sample wells, first add 40 µL of sample diluent, then add 10 µL of the sample (resulting in a final 5-fold dilution of the sample). Add samples to the bottom of the wells, avoiding contact with the well walls, and gently mix by pipetting.

d. Incubation: Cover the plate with a sealing membrane and incubate at 37 °C for 30 min.

e. Solution Preparation: Dilute the 20× concentrated wash buffer 20-fold with distilled water and set aside.

f. Washing: Carefully remove the sealing membrane, discard the liquid, and tap to remove excess liquid. Fill each well with wash buffer, let stand for 30 s, then discard. Repeat this process 5 times, then tap to dry.

g. Enzyme Addition: Add 50 µL of enzyme-labeled reagent to each well, excluding the blank well.

h. Incubation: Follow procedure 3.

i. Washing: Follow procedure 5.

j. Color Development: Add 50 µL of color developer A to each well, followed by 50 µL of color developer B. Gently mix by shaking. Incubate at 37 °C in the dark for 10 min.

k. Stop the reaction: Add 50 µL of stop solution to each well to terminate the reaction (the color will immediately change from blue to yellow).

l. Measure: Set the blank well to zero. Measure the absorbance (OD value) of each well sequentially at 450 nm. Measurements should be taken within 15 min after adding the stop solution.

Result Calculation: Plot a standard curve with the concentration of the standard substance on the *x*-axis and the OD value on the y-axis. Determine the corresponding concentration of the sample from the standard curve based on its OD value; then multiply by the dilution factor. Alternatively, calculate the linear regression equation of the standard curve using the concentrations and OD values of the standard substances. Substitute the sample’s OD value into the equation to calculate its concentration, then multiply by the dilution factor to obtain the actual concentration of the sample.

### 4.7. Volatile Metabolite Analysis

Remove samples from the −80 °C freezer and grind them in liquid nitrogen. Mix thoroughly using a vortex mixer. Weigh approximately 500 mg of each sample into headspace vials. Add 2 mL of saturated NaCl solution and 20 µL (10 µg/mL) of internal standard solution to each vial. Perform sample extraction using fully automated headspace solid-phase microextraction (HS-SPME) for GC-MS analysis.

Column: DB-5MS capillary column (30 m × 0.25 mm × 0.25 µm, Agilent J&W Scientific, Folsom, CA, USA); Injection port temperature: 250 °C; Injection volume: 5 µL. Flow rate: 1.2 mL/min; carrier gas: high-purity helium; temperature program: hold at 40 °C for 3.5 min, then increase to 100 °C at 10 °C/min, then to 180 °C at 7 °C/min, and finally to 280 °C at 25 °C/min, holding for 5 min.

Electron impact ion source (EI), ion source temperature 230 °C, quadrupole temperature 150 °C, mass spectrometer interface temperature 280 °C, electron energy 70 eV, scanning mode: selected ion monitoring (SIM), qualitative and quantitative ion precision scanning (GB 23200.8-2016) [38].

## 5. Conclusions

**Figure 11:**
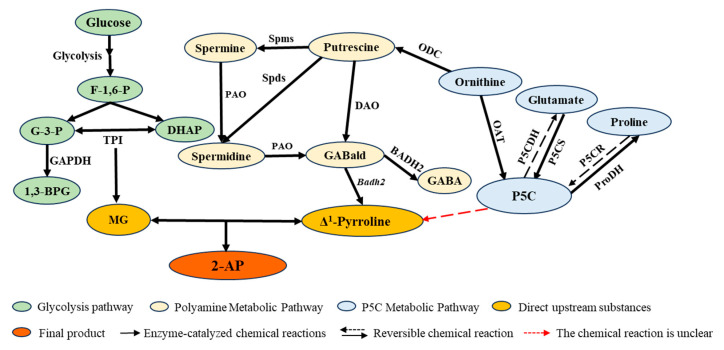
Synthesis Pathway of 2-AP. **Note:** The arrowheads indicate the corresponding enzymes; italics denote nonfunctional enzymes corresponding to mutated genes.

Based on previous studies, we have summarized the 2-AP synthetic metabolic pathway (Figure 11), which reveals the highly complex metabolic mechanism of 2-AP. Currently, the substances, genes, and enzyme activities involved in the proposed 2-AP metabolic pathway only exhibit differences between fragrant rice and non-fragrant rice. Among fragrant rice varieties, only the expression level of *OsBadh2* and GABA content show a negative correlation with 2-AP content. Upstream gene expression levels, specific enzyme activities, and substance contents all exhibit non-linear relationships with 2-AP content. This study suggests that 2-AP synthesis relies more on critical nodes (*OsBadh2* deficiency) than on a linear increase in overall metabolic flux. Therefore, intensifying research on the mechanisms by which 2-AP metabolic pathways influence 2-AP content in fragrant rice, and identifying key metabolites and genes regulating 2-AP levels within these pathways, will facilitate the targeted enhancement of grain 2-AP content. Currently, beyond *OsBadh2*, the metabolic basis of aroma remains elusive, and the genetic foundation for the accumulation of aromatic metabolites is largely unknown. Identifying new genes regulating aromatic 2-AP will represent a major breakthrough in aromatic rice breeding.

## Figures and Tables

**Table 1:** Sequence alignment of rice *OsBadh2* nucleic acid.

Type	Accession	Badh2-Exon7(+727~+747)
Non-Fragrant	Nipponbare	GGTAAAAAGATTATGGCTTCA
Fragrant	YZX	GGTATATA - - - - - - - - TTTCA
Fragrant	XYXZ	GGTATATA - - - - - - - - TTTCA
Fragrant	DT502	GGTATATA - - - - - - - - TTTCA
Fragrant	DLX	GGTATATA - - - - - - - - TTTCA
Fragrant	XG6	GGTATATA - - - - - - - - TTTCA
Fragrant	XG12	GGTATATA - - - - - - - - TTTCA

**Note:** Red text indicates base mutations; “-” indicates base deletions.

**Table 2:** Nucleic acid sequence alignment of rice *OsPAO4*.

Type	Accession	+551~+560
Non-Fragrant	Nipponbare	AATGGTGTGT
Fragrant	YZX	AATGGTGTGT
Fragrant	XYXZ	AATGGTGTGT
Fragrant	DT502	AATGGTGTGT
Fragrant	DLX	AATGGTGTGT
Fragrant	XG6	AATGGCGTGT
Fragrant	XG12	AATGGTGTGT

**Note:** Red text indicates base mutations.

**Table 3:** Nucleic acid sequence alignment of rice *OsP5CS1*.

*OsP5CS1* (CDS)								
Type	Accession	+191~+200	+881~+890	+921~+930	+946~+955	+1651~+1660	+1671~+1680	+1686~+1695
Non-Fragrant	9311	TCCATATGAG	GTACTCGTGA	GCATCTACAG	GAACGAAAAA	GTTCATAAGG	GAGTCCAGGC	TATTAGTAGC
Fragrant	YZX	TCCATATGAG	GTGCTCGTGA	GCGTCTACAG	GAACGGAAAA	GTTCATAAGG	GAATCCAGGC	TATTAGTAGC
Fragrant	XYXZ	TCCATATGAG	GTGCTCGTGA	GCGTCTACAG	GAACGGAAAA	GTTCATAAGG	GAATCCAGGC	TATTAGTAGC
Fragrant	DT502	TTTCATATGAG	GTGCTCGTGA	GCGTCTACAG	GAACGGAAAA	GTTCATAAGG	GAATCCAGGC	TATTAGTAGC
Fragrant	DLX	TCCATATGAG	GTACTCGTGA	GCATCTACAG	GAACGAAAAA	GTTCATAAGG	GAGTCCAGGC	TATTAGTAGC
Fragrant	XG6	TCCATATGAG	GTGCTCGTGA	GCGTCTACAG	GAACGGAAAA	GATCATAAGG	GAATCCAGGC	TATTAGTAGC
Fragrant	XG12	TCCATATGAG	GTACTCGTGA	GCATCTACAG	GAACGAAAAA	GTTCATAAGG	GAGTCCAGGC	TATTAGCAGC

**Note:** Red text indicates base mutations.

**Table 4:** Information on test varieties.

Material Name	Male Parent	Famale Parent	Breeding Unit
YZX	TLX103	R4015	Hunan Rice Research Institute; Hunan Jinjian Rice Industry Co. (Changde, China)
XYXZ	XYXZ	YZX	Hunan Golden Rice Seed Industry Co. (Changsha, China)
DT502	DQ20	HP	Seed Management Station of Indochina, Yunnan Province; Yunnan Type Hybrid Rice Institute, Yunnan Province
DLX	γ-194	DLXD	Guizhou Rice Resrarch Institute
XG6	Selection of a mutant strain of Xiligongmi		Guizhou Rongjiang Shengtai Agricultural Products Development Co.
XG12	Selection of a mutant strain of Xiligongmi		Guizhou Rongjiang Shengtai Agricultural Products Development Co.

**Table 5:** Key genes in the 2-AP synthesis pathway.

Gene Name	Gene Symbol	Locus ID
Diamine oxidase	*OsDAO4*	LOC_Os04g40040
Polyamine oxidase	*OsPAO4*	LOC_Os04g57550
Ornithine decarboxylase	*OsODC*	LOC_Os02g28110
Ornithine aminotransferase	*OsOAT*	LOC_Os03g44150
Proline dehydrogenase	*OsProDH*	LOC_Os10g40360
Δ^1^-pyrroline-5-carboxylate synthetase	*OsP5CS1*	LOC_Os05g38150
Δ^1^-pyrroline-5-carboxylate synthetase	*OsP5CS2*	LOC_Os01g62900

## Data Availability

The data from this study are available for sharing. Researchers with a legitimate need may contact the corresponding author to obtain the data.
